# Long-Term Outcomes of a Surgical Technique in Management of Subconjunctival Orbital Fat Prolapse

**DOI:** 10.7759/cureus.6078

**Published:** 2019-11-05

**Authors:** Ken-ichi Sato

**Affiliations:** 1 Ophthalmology, Nikko Memorial Hospital, Muroran, JPN

**Keywords:** adipose tissue, hernia, operative procedures, orbit

## Abstract

Objective

This retrospective, single-center, interventional study presented the long-term results of a novel surgical technique for the management of subconjunctival orbital fat prolapse.

Methods

Nine eyes of seven consecutive patients were surgically repaired using the technique involving connective tissue repair and were intended to be followed-up for more than five years.

Results

All surgeries performed were uneventful and esthetic enhancement was achieved for each patient. No postoperative complications were noted. There was no recurrence throughout the follow-up period; six eyes of four patients were followed-up for more than five years.

Conclusions

This technique with connective tissue repair achieved good long-term results.

## Introduction

Subconjunctival orbital fat prolapse is a rare benign condition characterized by a yellowish mobile mass just below the bulbar conjunctiva, typically in the superotemporal quadrant. Patients are mostly male [1-4]. Recently, several surgical techniques for this disorder have been described, some of which are advocated as being minimally invasive [5-9]. However, knowledge of long-term results after such surgeries is still insufficient [8,10].

A preliminary study of four cases presented a surgical technique for the management of subconjunctival orbital fat prolapse, attempting to avoid recurrence through connective tissue repair [11]. The study also revealed the source of the prolapsed fat as being intraconal fat. This paper reports the long-term outcomes of the cases described in the preliminary study, with three additional patients.

## Materials and methods

This was a retrospective, single-center, interventional study. Included in the study were nine eyes of seven consecutive patients who underwent surgery for subconjunctival orbital fat prolapse at Nikko Memorial Hospital from December 2003 to October 2009 (Table 1), and were intended to be followed-up for more than five years; four cases were reported on in a preliminary study [11]. 

**Table 1:** Patients who underwent surgery for subconjunctival orbital fat prolapse.

Age at surgery (years) /Sex	Laterality	Follow-up period (years)	Reason for shortfollow-up period
67/Male	Bilateral	13.8	-
62/Male	Left	12.6	-
69/Male	Bilateral	7.3	-
87/Male	Left	1.0	Died
79/Male	Left	1.3	Follow-up defaulted
70/Male	Right	0.6	Follow-up defaulted
69/Male	Left	7.9	-

**Figure 1:**
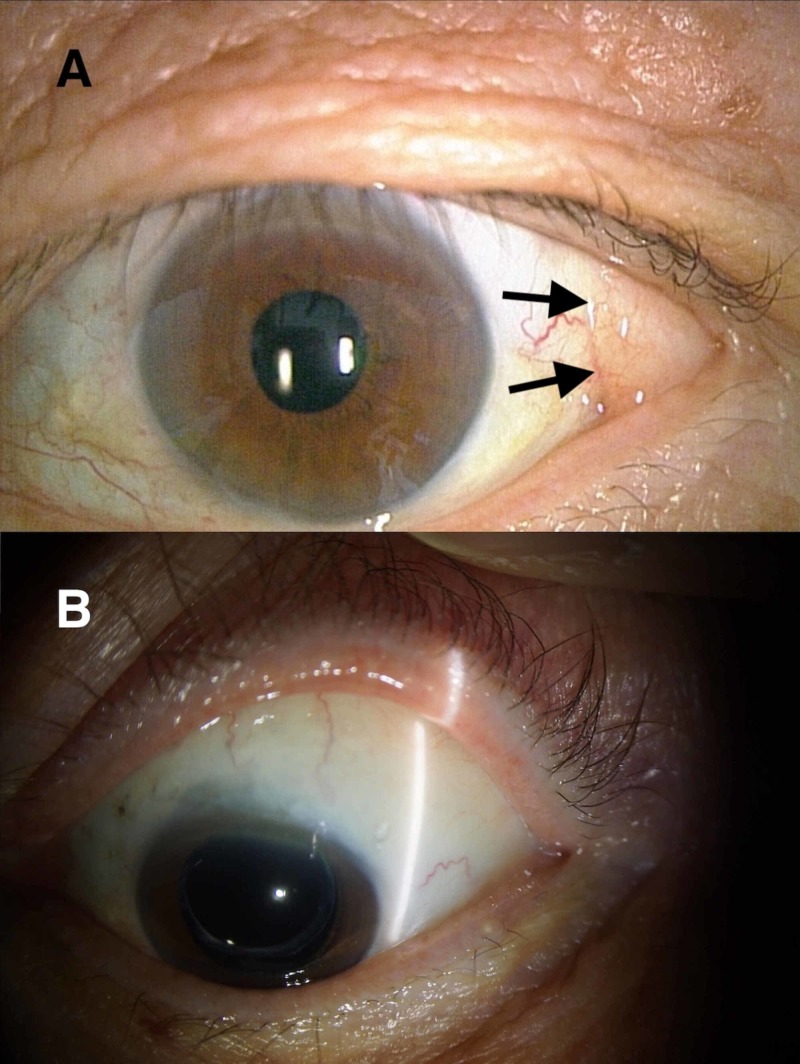
Prolapsed subconjunctival fat in the superotemporal quadrant of the left eye of a man. (A) Preoperative findings (69 years old). (B) Findings at 7.9 years postoperatively.

The patients all had fat prolapse in the superotemporal quadrant with no history of trauma or surgery (Figure 1A), and had a clinical diagnosis based on the report by Glover and Grove [1].

Written consent was obtained from all patients before surgery. All procedures performed in this study were in accordance with the ethical standards of the institutional research committee and with the 1964 Helsinki declaration and its later amendments or comparable ethical standards.

Details of the surgical technique (Video 1) have been fully described in the preliminary study [11].

**Video 1:** A surgical technique with connective tissue repair in the management of subconjunctival orbital fat prolapse.

**Figure 2:**
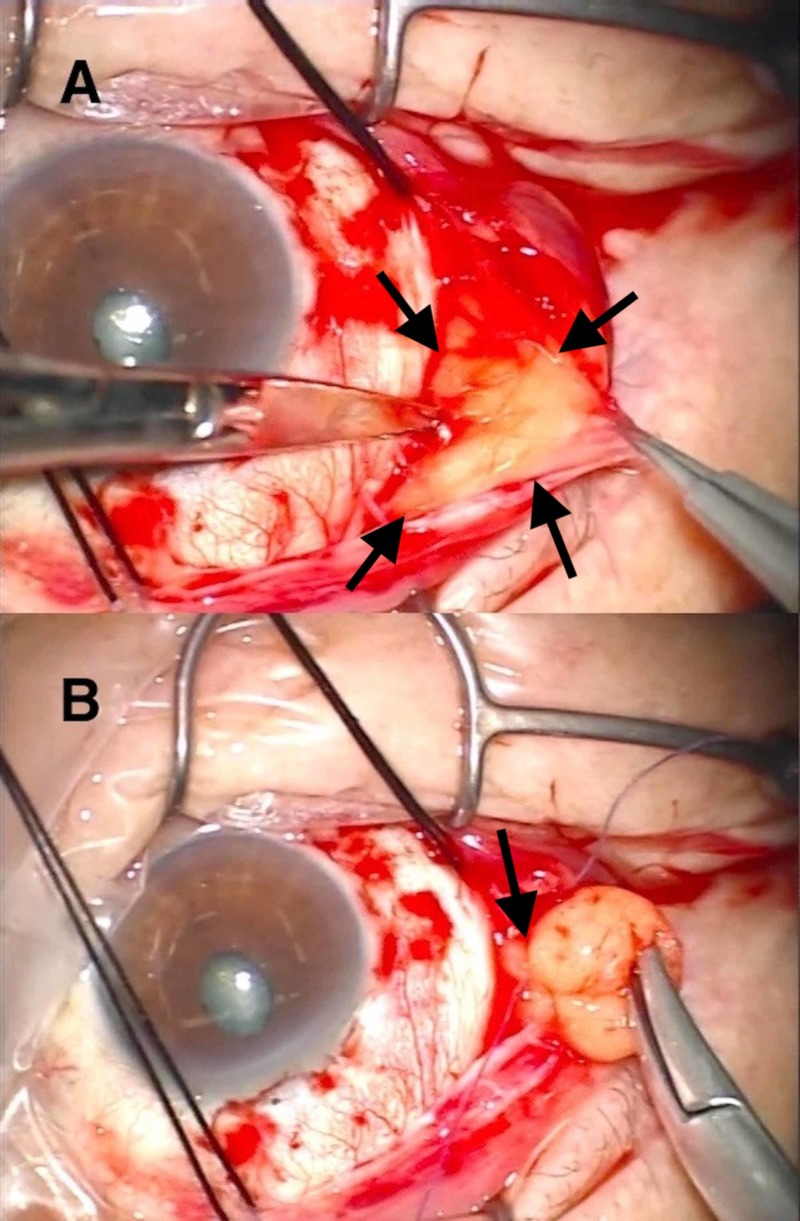
Perspective of the surgeon. Right eye of a 70-year-old man. (A) Fat (arrows) is extruded by blunt separation of connective tissue covering the inner surface of the fat. Note that the connective tissue is everted using forceps. (B) The base of the extruded fat is ligated (arrow).

Briefly, a 120° fornix-based conjunctival incision centered superotemporally was made under local anesthesia. The adhesion of Tenon’s capsule to the limbal episclera was dissected, and the Tenon’s capsule anterior to the equator was separated from the episclera. The sheet of connective tissue subsequently formed by the above procedure was everted, and the connective tissue covering the inner surface of the herniated fat was bluntly dissected to allow extrusion of the fat (Figure 2A).

The base of the extruded fat was ligated using a 6-0 polyglactin suture (Vicryl; Ethicon Inc., Somerville, NJ, USA) to create a pedunculated appearance (Figure 2B). Using the same ligature, the herniated fat was fixed to the episclera, 6-8 mm posterior to the limbus, after resection of the fat distal to the ligature. The connective tissue sheet was then repositioned on the episclera with several 6-0 polyglactin sutures, just anterior to the fixation of the fat, and the conjunctiva was subsequently closed. 

Outcome measures were intra- and postoperative complications, satisfactory cosmetic results, and recurrence.

## Results

Six eyes in four cases were followed up for more than five years (Table 1). Each surgery was uneventful and esthetic enhancement was achieved (Figure 1B). There were no postoperative complications, such as symptoms of dry-eye or impaired ocular motility due to likely muscle restriction. Also, the herniated fat was not observed in the primary eye position in all the cases throughout the follow-up period.

## Discussion

The preliminary study demonstrated that the spontaneous prolapsed fat at the superior temporal quadrant stems from intraconal fat, and therefore, the anterior part of the herniated fat is covered by both the intermuscular septum and Tenon’s capsule [11]. The novel technique reported in the study attempted to avoid recurrence via reconstruction of the intermuscular septae. In addition, the previous study reported initial results. The present study reports the long-term outcomes.

Although the technique is relatively complicated and invasive, it is expected to prevent recurrence better than less invasive techniques that have been previously reported (Table 2), through the added procedure of connective tissue repair [5-9]. In the present study, no recurrence occurred, even in the six eyes with observation periods of more than five years; this potentially demonstrates the robust efficacy of the technique.

**Table 2:** Reports of less invasive techniques for the management of orbital fat prolapse.

Author	Year	Country	Sample (eyes/patients)	Suture	Incision	Mean follow-up period (years)	Recurrence (eye[s])
Otaka et al. [5]	2001	Japan	4/3	+	-	1.0	0
Sniegowski et al. [6]	2012	U.S.A.	4/3	-	+	2.6	0
Nakamura et al. [7]	2015	Japan	23/19	+	-	1.6	1
Yang et al. [8]	2017	Korea	48/37	+	+	3.3	2
Raparia et al. [9]	2018	U.S.A.	68/45	-	+	3.1	0

This study had certain limitations. The number of patients was small and, as mentioned above, the technique is relatively invasive; the results of this technique should therefore be compared with outcomes involving less-invasive techniques in a larger study.

## Conclusions

The long-term outcomes of a newly developed surgical technique with connective tissue repair for the management of subconjunctival orbital fat prolapse was presented. All subjects achieved cosmetic improvement without complications or recurrence.
